# Interacting and joint effects of triglyceride-glucose index and hypertension on stroke risk in middle-aged and older Chinese adults: a population-based prospective cohort study

**DOI:** 10.3389/fcvm.2024.1363049

**Published:** 2024-05-15

**Authors:** Yun-Dan Luo, Ying-Yuan Gan, Qian Liao, Xu Li, Rong-Rui Huo

**Affiliations:** ^1^ Department of General Practice, Minzu Hospital of Guangxi Zhuang Autonomous Region, Nanning, China; ^2^ Department of Scientific Research, Minzu Hospital of Guangxi Zhuang Autonomous Region, Nanning, China; ^3^ Department of Epidemiology and Health Statistics, School of Public Health, Guangxi Medical University, Nanning, China; ^4^ Guangxi Health Commission Key Laboratory of Clinical Biotechnology, Liuzhou People’s Hospital, Liuzhou, China; ^5^ Department of Experimental Research, Guangxi Medical University Cancer Hospital, Nanning, China

**Keywords:** stroke, triglyceride-glucose index, hypertension, insulin resistance, interactive effects

## Abstract

**Background:**

Triglyceride-glucose (TyG) index and hypertension were well-established risk factors for stroke. And TyG index was associated with hypertension. However, no prior study has investigated the interactive effects of the TyG index and hypertension on stroke. This study examined whether hypertension mediates associations of TyG index with incident stroke and the extent of interaction or joint relations of TyG index and hypertension with stroke in middle-aged and older Chinese adults.

**Methods:**

The China Health and Retirement Longitudinal Study (CHARLS) is an ongoing nationally representative prospective cohort study initiated in 2011. This cohort study included 9,145 middle-aged and older Chinese adults without stroke at baseline. The eposures were TyG index and the logarithmized product of hypertension, as determined during the baseline health examination. The main outcome was self-reported physician-diagnosed stroke which followed up from June 1, 2011, to June 30, 2018.

**Results:**

Of the 9,145 participants, 4,251 were men (46.5%); the mean (SD) age was 59.20 (9.33) years. During a median follow-up of 7.1 years, 637 (7.0%) participants developed stroke. In multivariable-adjusted models, the TyG index was significantly associated with the risk of hypertension [odds ratio (OR) per 1-SD increase, 1.29; 95% CI, 1.19–1.41] and stroke [hazard ratio (HR) per 1-SD increase, 1.16; 95% CI, 1.02–1.33]. Both multiplicative and additive interactions were observed between TyG index and hypertension on stroke (HR for multiplicative: 2.34, 95% CI, 1.57–3.48; Synergy index: 4.13, 95% CI, 2.73–6.25). Mediation analysis showed that 20.0% of the association between TyG index and stroke was mediated through hypertension.

**Conclusions:**

This study suggests a synergistic effect of TyG index and hypertension on stroke, and a small proportion of the association between TyG index and stroke was mediated by hypertension, indicating the benefit of coordinated control strategies for both exposures.

## 1 Introduction

Stroke continues to be one of the major causes of mortality and long-term disability globally (1, 2). A comprehensive understanding of its multifactorial etiology is crucial for improving prevention and treatment (1, 2). Among the range of risk factors that contribute to stroke, hypertension remains an established modifiable element, with a global prevalence that demands attention (3, 4). Simultaneously, emerging research has increasingly underscored the importance of metabolic indices for cardiovascular health. The triglyceride-glucose (TyG) index, a reliable measure of insulin resistance (5, 6), has been particularly highlighted as a significant cardiometabolic risk indicator (7).

The role of hypertension in elevating the risk of stroke has been well-documented (4, 8). It has a linear relationship with both ischemic and hemorrhagic strokes, mediated through mechanisms such as arterial wall thickening, endothelial dysfunction, and increased inflammatory responses (8, 9). These understandings have informed the critical practice of managing blood pressure as a strategy for mitigating stroke risk. In parallel, the TyG index has gained substantial scientific focus due to its low-cost and reliable representation of insulin resistance (10, 11). It is associated with a variety of cardiovascular diseases and has been specifically linked to hypertension (12–15). This interconnected pathway between the TyG index and hypertension indicates a potential mechanism that could exacerbate stroke risk. Although research has adequately addressed the individual relationships between the TyG index and hypertension with stroke, a notable gap exists in the literature concerning their interactive or joint contributions to stroke risk. This gap in research makes it evident that a detailed examination of the possible mediation and interaction between these two factors is necessary. Such an examination would not only deepen the understanding of stroke's etiology but also refine risk stratification and potentially inform new treatment guidelines considering multiple interacting risk factors.

To fill this knowledge gap, the objective of the current study is to investigate whether hypertension mediates the associations of the TyG index with incident stroke. The study will also quantify the extent of interaction or joint relations between the TyG index and hypertension concerning stroke risk. This exploration aims to add valuable insights into stroke's multifactorial risk factors, ultimately enabling clinicians to implement more focused and comprehensive preventive measures.

## 2 Methods

### 2.1 Study design and population

This study presents a secondary analysis of data sourced from CHARLS, a comprehensive nationwide cohort study designed to represent the national population (16). The design and methodology of CHARLS have been elaborated in previous publications (16). Succinctly, the study sampled 17,708 participants from 10,257 households, utilizing a multistage stratified probability-proportional-to-size sampling method. Participants were enlisted from 150 counties or districts and 450 villages across 28 provinces in China between June 2011 and March 2012. Using a standardized questionnaire, data on sociodemographic, lifestyle factors, and health-related aspects were collected. The initial survey, Wave 1, achieved an 80.5% response rate. Follow-up assessments were conducted biennially: Wave 2 in 2013, Wave 3 in 2015, and Wave 4 in 2018. For the current analysis, only participants aged 45 or above with comprehensive data on fasting blood glucose (FBG), triglycerides (TG), hypertension, and blood pressure were considered. Those with a prior history of stroke were excluded.

The CHARLS study secured approval from Peking University's institutional review board, and all participants provided written informed consent. The current study is compliant with the Strengthening the Reporting of Observational Studies in Epidemiology (STROBE) guidelines (17).

### 2.2 Assessment of TyG index and hypertension

The Beijing-based Chinese Center for Disease Control and Prevention swiftly received venous blood samples within two weeks of their dispatch from the CDC station. These samples were promptly preserved at −20°C until transport. Post assay completion at the Chinese Medical University laboratory, they were relocated to a deep freezer, with a maintained temperature of −80°C. TG and FPG concentrations were analyzed using the enzyme colorimetric assay at the Youanmen Clinical Laboratory of Capital Medical University. The TyG index was computed using the formula ln [TG (mg/dl) × FPG (mg/dl)/2] (18).

Hypertension was characterized by a systolic blood pressure of ≥140 mmHg, diastolic blood pressure of ≥90 mmHg, ongoing antihypertensive medication use, or a self-reported history of the condition. Blood pressure measurements were undertaken by trained nursing personnel.

### 2.3 Outcome ascertainment

The primary outcome of this study was the incidence of stroke. In accordance with previous studies (19, 20), stroke incidents were identified through self-reports wherein participants confirmed a physician's diagnosis of stroke. The recorded date of the stroke diagnosis was demarcated as the interval between the date of the latest interview and the date when the incident stroke was reported (19, 20).

### 2.4 Covariates

During the initial assessment at Wave 1, trained interviewers gathered sociodemographic and health-related data via a structured questionnaire, including age, gender, residence, marital status, and educational level (categorized as no formal education, primary school, middle or high school, or college and above). Marital status was classified as either married or another marital status (including individuals who were never married, separated, divorced, or widowed). Health-related variables included self-reported smoking and drinking status (categorized as never, former, or current), self-reported physician-diagnosed medical conditions (diabetes, heart disease, kidney disease, and dyslipidemia), and the use of medications for diabetes, hypertension, and dyslipidemia. Laboratory tests included measurements of total cholesterol (TC), high-density lipoprotein cholesterol (HDL-C), low-density lipoprotein cholesterol (LDL-C), estimated glomerular filtration rate (eGFR), and glycosylated hemoglobin, type A1c (HbA1c) (21). Diabetes was defined as fasting plasma glucose ≥126 mg/dl, current use of antidiabetic medication, or self-reported history of diabetes. Dyslipidemia was defined as total cholesterol levels ≥240 mg/dl, triglyceride levels ≥150 mg/dl, low-density lipoprotein cholesterol levels ≥160 mg/dl, high-density lipoprotein cholesterol levels <40 mg/dl, current use of lipid-lowering medication, or self-reported history of dyslipidemia. Height and weight were measured by a trained nurse. Body mass index was calculated as weight in kilograms divided by height in meters squared. The eGFR was calculated using the Chronic Kidney Disease Epidemiology Collaboration's 2009 creatinine equation (22).

### 2.5 Statistical analysis

Statistical analysis was conducted from October 1, 2023, to October 23, 2023. Data were described as medians and interquartile ranges for continuous variables. The frequency with percentage was used to describe categorical variables. The *χ*^2^ test and Kruskal-Wallis rank sum test were utilized to compare differences in the baseline characteristics between the different groups, as appropriate. Five percent (452 of 9,145) of total data items were missing, were assumed to be missing at random, and, thus, were imputed with the multiple imputation of chained equations method using the baseline characteristics.

We used Fine and Gray competing risk models (23) to estimate the hazard ratios (HRs) and 95% confidence intervals (CIs) of stroke associated with hypertension, systolic blood pressure (categorized as quartiles), diastolic blood pressure (categorized as quartiles), and TyG index (categorized as quartiles); we also used logistic regression models to estimate the odds ratios (ORs) and 95% CIs of hypertension associated with TyG index. Three models were estimated: in model 1, age and sex were adjusted; in model 2, age, sex, residence, marital status, educational level, body mass index, smoking status, and drinking status were adjusted; and in model 3, the variables in model 2 plus history of diabetes, heart disease, dyslipidemia, kidney disease, history of medication use for diabetes, history of medication use for dyslipidemia, TC, HDL-C, LDL-C, HbA1c, hsCRP, and eGFR were adjusted. In addition, we explored the potential nonlinear associations using 4-knotted restricted cubic spline (RCS) regression, and the models were conducted with 4 knots at the 5th, 35th, 65th, and 95th percentiles of TyG index, systolic blood pressure, or diastolic blood pressure (reference is the 5th percentile).

To quantify the additive and multiplicative interactions, we additionally included a product term of blood pressure (hypertension, systolic blood pressure, or diastolic blood pressure) and TyG index (quartiles) in the model. The HR with its 95% CIs of the product term was the measure of interaction on the multiplicative scale, and the multiplicative interaction was statistically significant when its CIs did not include 1. We assessed additive interactive effects using the synergy index (SI). This metric captures different aspects of interaction, including the part of the effect attributable to interaction, the proportion of the combined effect arising from interaction, and the ratio between the combined effect and individual effects. Specifically, SI = 1 indicates the absence of interactive effects between blood pressure and TyG index concerning stroke incidence. Conversely, when SI > 1, this signifies that the combined effects of blood pressure and TyG index on stroke incidence exceed the sum of their individual effects, suggesting synergistic effects. Conversely, if SI < 1, it indicates that the combined effects are smaller than the sum of the individual effects of blood pressure and TyG index. We computed the corresponding 95% CIs for SI using the delta method, and the additive interaction was statistically significant when its CIs did not include 1.

We proceeded to assess indirect associations of TyG index with stroke mediated by hypertension and direct associations unmediated by blood pressure (hypertension, systolic blood pressure, or diastolic blood pressure). Using the “mediation” package in R, we applied a regression-based approach to compute the total effect, natural indirect effects (NIE), and natural direct effects (NDE) of the TyG index on stroke incidence (Supplementary Figure S1). Two models were constructed: one involved a multivariable logistic regression model for blood pressure (mediator), conditioned on TyG index (exposure) and confounders, while the other encompassed a multivariable Cox proportional hazard regression model for stroke (outcome), conditioned on TyG index, blood pressure, and confounders. NDE conveyed the impact the of TyG index on stroke independent of blood pressure, whereas NIE represented the proportion of TyG index influenced by its connection with blood pressure over time. To gauge the extent of mediation, we computed the proportion of the association mediated by TyG as NIE divided by the sum of NDE and NIE.

To test the robustness and potential variations in different subgroups, we repeated all analyses stratified by gender (male and female), and age groups (<60 years, and ≥60 years, defined as elders by the World Health Organization (24). And, sensitivity analyses were conducted as follows: (1) repeating all analyses using the complete data set (8,693 participants) without multiple imputations; (2) using blood pressure (hypertension, systolic blood pressure, or diastolic blood pressure) at Wave 2 and excluded participants who have hypertension at baseline when analyzed their mediating effects to minimize the possibility of reverse causality on the observed associations. All analyses were performed using R statistical software version 4.3.0 (R Foundation). We considered two-sided *P* values <0.05 to be significant.

## 3 Results

### 3.1 Population characteristics

Of the 17,708 CHARLS participants at study baseline, we excluded 777 individuals younger than 45 years, 634 with stroke at baseline, 5,471 missing TyG index, 643 missing systole blood pressure, 2 missing diastolic blood pressure, and 36 missing hypertension. Finally, 9,145 participants were included for analysis. A detailed description of the selection process for the study analytic sample is included in Supplementary Figure S2. A comparison of baseline characteristics between participants included and those who were not included in the analysis is shown in Supplementary Table S1.

The mean (SD) age at baseline was 59.20 (9.33) years; 4,251 (46.5%) of the participants were men. Table 1 shows the characteristics of the participants. At baseline, the mean (SD) TyG index was 8.67 (0.68), the mean (SD) systole blood pressure was 129.59 (21.50) mmHg, while the mean (SD) diastolic blood pressure was 75.32 (12.23) mmHg (Supplementary Figure S3–S4), and 41.1% (3,757/9,145) participants experienced hypertension. When compared with Q1 of TyG index, participants in the other quartiles were more likely to be female, body mass index ≥28.0 kg/m^2^ and married; to reside in an urban setting; to have no formal education, fewer current smokers and drinkers, and higher systolic and diastolic blood pressure; to have a higher prevalence of hypertension, diabetes, dyslipidaemia, and heart disease; to have a history of medication use for diabetes and dyslipidemia; to have higher TC, LDL-C HbA1, hsCRP, and eGFR; and to have lower HDL-C levels (Table 1).

**Table 1:** Characteristics of the participants according to TyG index.

Characteristic	Overall	TyG index				*P* value*
		Q1 [5.18, 8.23]	Q2 (8.23, 8.60]	Q3 (8.60, 9.05]	Q4 (9.05, 13.00]	
*n*	9,145	2,288	2,285	2,286	2,286	
Age, years						0.262
<60	5,096 (55.7%)	1,279 (55.9%)	1,255 (54.9%)	1,251 (54.7%)	1,311 (57.3%)	
≥60	4,049 (44.3%)	1,009 (44.1%)	1,030 (45.1%)	1,035 (45.3%)	975 (42.7%)	
Gender						<0.001
Male	4,251 (46.5%)	1,256 (54.9%)	1,082 (47.4%)	957 (41.9%)	956 (41.8%)	
Female	4,894 (53.5%)	1,032 (45.1%)	1,203 (52.6%)	1,329 (58.1%)	1,330 (58.2%)	
Marital status						0.014
Married	7,632 (83.5%)	1,910 (83.5%)	1,899 (83.1%)	1,871 (81.8%)	1,952 (85.4%)	
Other	1,513 (16.5%)	378 (16.5%)	386 (16.9%)	415 (18.2%)	334 (14.6%)	
Residence						<0.001
Urban	3,199 (35.0%)	675 (29.5%)	754 (33.0%)	832 (36.4%)	938 (41.0%)	
Rural	5,946 (65.0%)	1,613 (70.5%)	1,531 (67.0%)	1,454 (63.6%)	1,348 (59.0%)	
Education level						0.005
No formal education	2,720 (29.7%)	661 (28.9%)	673 (29.5%)	732 (32.0%)	654 (28.6%)	
Primary school	3,725 (40.7%)	960 (42.0%)	956 (41.8%)	896 (39.2%)	913 (39.9%)	
Middle or high school	2,425 (26.5%)	609 (26.6%)	588 (25.7%)	603 (26.4%)	625 (27.3%)	
College or above	275 (3.0%)	58 (2.5%)	68 (3.0%)	55 (2.4%)	94 (4.1%)	
Body mass index, kg/m^2^^a^						<0.001
<18.5	625 (6.8%)	237 (10.4%)	201 (8.8%)	131 (5.7%)	56 (2.4%)	
18.5–23.9	4,730 (51.7%)	1,474 (64.4%)	1,292 (56.5%)	1,097 (48.0%)	867 (37.9%)	
24.0–27.9	2,639 (28.9%)	446 (19.5%)	580 (25.4%)	749 (32.8%)	864 (37.8%)	
≥28.0	1,034 (11.3%)	105 (4.6%)	181 (7.9%)	289 (12.6%)	459 (20.1%)	
Smoking status^a^						<0.001
Never	5,552 (60.7%)	1,275 (55.7%)	1,371 (60.0%)	1,439 (62.9%)	1,467 (64.2%)	
Former	794 (8.7%)	205 (9.0%)	179 (7.8%)	200 (8.7%)	210 (9.2%)	
Current	2,772 (30.3%)	801 (35.0%)	727 (31.8%)	638 (27.9%)	606 (26.5%)	
Drinking status^a^						<0.001
Never	5,393 (59.0%)	1,240 (54.2%)	1,321 (57.8%)	1,421 (62.2%)	1,411 (61.7%)	
Former	752 (8.2%)	173 (7.6%)	204 (8.9%)	206 (9.0%)	169 (7.4%)	
Current	2,995 (32.8%)	874 (38.2%)	759 (33.2%)	657 (28.7%)	705 (30.8%)	
History of comorbidities						
Diabetes^a^	555 (6.1%)	47 (2.1%)	71 (3.1%)	122 (5.3%)	315 (13.8%)	<0.001
Heart disease^a^	1,074 (11.7%)	193 (8.4%)	245 (10.7%)	273 (11.9%)	363 (15.9%)	<0.001
Dyslipidemia^a^	863 (9.4%)	110 (4.8%)	157 (6.9%)	225 (9.8%)	371 (16.2%)	<0.001
Kidney disease^a^	540 (5.9%)	135 (5.9%)	145 (6.3%)	137 (6.0%)	123 (5.4%)	0.574
History of medication use						
Diabetes medications^a^	351 (3.8%)	26 (1.1%)	43 (1.9%)	73 (3.2%)	209 (9.1%)	<0.001
Dyslipidemia medications^a^	447 (4.9%)	51 (2.2%)	78 (3.4%)	115 (5.0%)	203 (8.9%)	<0.001
Median TC (IQR), mg/dl	190.59 (167.40, 215.72)	176.29 (156.19, 199.49)	188.66 (166.62, 210.70)	194.85 (172.04, 219.20)	204.51 (179.00, 234.28)	<0.001
Median HDL-C (IQR), mg/dl^a^	49.48 (40.59, 59.92)	58.76 (50.26, 68.43)	52.96 (45.23, 62.63)	47.17 (40.21, 55.67)	39.82 (32.86, 47.55)	<0.001
Median LDL-C (IQR), mg/dl^a^	114.43 (93.56, 137.24)	106.32 (88.92, 126.13)	116.37 (97.42, 137.63)	121.39 (100.52, 143.82)	113.66 (87.76, 141.50)	<0.001
Median HbA1c (IQR), %^a^	5.10 (4.90, 5.40)	5.00 (4.80, 5.30)	5.10 (4.80, 5.40)	5.10 (4.90, 5.40)	5.30 (5.00, 5.80)	<0.001
Median hsCRP (IQR), mg/L	1.04 (0.55, 2.18)	0.81 (0.46, 1.83)	0.91 (0.51, 1.97)	1.07 (0.60, 2.20)	1.32 (0.71, 2.73)	<0.001
Median eGFR (IQR), ml/min/1.73 m^2^^a^	72.78 (53.41, 95.86)	71.64 (53.20, 93.43)	72.46 (52.09, 94.62)	72.67 (52.18, 96.36)	74.77 (56.09, 99.82)	<0.001
Median TG (IQR), mg/dl	105.32 (75.22, 153.99)	61.06 (52.22, 69.92)	90.27 (80.54, 100.89)	128.32 (113.28, 144.26)	208.86 (172.57, 277.89)	<0.001
Median FBG (IQR), mg/dl	102.42 (94.50, 113.76)	95.22 (88.38, 102.60)	99.90 (93.60, 107.28)	103.86 (96.48, 113.22)	116.28 (104.58, 143.46)	<0.001
Median TyG index (IQR)^b^	8.60 (8.23, 9.05)	7.99 (7.82, 8.12)	8.42 (8.33, 8.51)	8.80 (8.70, 8.92)	9.43 (9.20, 9.80)	<0.001
Median systole blood pressure (IQR), mmHg	126.50 (114.00, 141.50)	122.50 (110.50, 137.50)	124.50 (112.50, 139.50)	128.50 (115.50, 142.50)	130.50 (117.62, 145.00)	<0.001
Median diastolic blood pressure (IQR), mmHg	74.50 (67.00, 83.00)	72.00 (64.50, 80.50)	74.00 (66.50, 82.00)	75.00 (67.50, 83.50)	77.00 (69.00, 85.00)	<0.001
Hypertension	3,757 (41.1%)	722 (31.6%)	818 (35.8%)	1,022 (44.7%)	1,195 (52.3%)	<0.001
Stroke	637 (7.0%)	101 (4.4%)	144 (6.3%)	198 (8.7%)	194 (8.5%)	<0.001

Data are presented as mean ± SD or *n* (%), unless otherwise specified.

eGFR, estimated glomerular filtration ratio; FBG, fasting blood glucose; HbA1c, glycated hemoglobin; HDL-C, high-density lipoprotein cholesterol; IQR, interquartile range; LDL-C, low-density lipoprotein cholesterol; TC, total cholesterol; TG, triglyceride; TyG, triglyceride-glucose.

^a^ Missing data: 117 for body mass index, 27 for smoking status, 5 for drinking status, 77 for diabetes, 28 for heart disease, 159 for dyslipidemia, 39 for kidney disease, 78 for history of medication use for diabetes, 163 for history of medication use for dyslipidemia, 2 for HDL-C, 17 for LDL-C, 70 for HbA1c, 4 for eGFR.

^b^ The TyG index was calculated by the formula ln [TG (mg/dl) × FBG (mg/dl)/2].

* *P* value was based on *χ*^2^ or Kruskal-Wallis rank sum test where appropriate.

### 3.2 Association of TyG index and hypertension with stroke

During a median follow-up of 7.1 years, 637 (7.0%) participants experienced an incident stroke. Table 2 shows the associations between TyG index and incident stroke. After adjusting for confounders (in model 3), when compared with Quartile 1, the adjusted HRs (95% CIs) for incident stroke were 1.27 (0.98–1.65) for Quartile 2, 1.55 (1.19–2.02) for Quartile 3, and 1.21 (0.88–1.66) for Quartile 4. A one SD increase in TyG index was associated with an elevated risk of stroke (HR, 1.16; 95% CI, 1.02–1.33). A nonlinear association between the TyG index and risk of incident stroke using RCS regression was also found (for association, *P* = 0.004; for nonlinear, *P* = 0.009; Supplementary Figure S5A). Hypertension, systole blood pressure ≥140 mmHg, and diastolic blood pressure ≥90 mmHg were independently associated with increased risk of incident stroke, the HRs were 2.10 (95% CI, 1.76–2.50), 1.62 (95% CI, 1.37–1.92), and 1.70 (95% CI, 1.39–2.08), respectively. Similar results were found when modeling the systole blood pressure and diastolic blood pressure as quartiles, and a linear and positive association of systole blood pressure and diastolic blood pressure with the risk of incident stroke using RCS regression were also found (Supplementary Figures S5B,C). Similar results were found when complete data analyses were conducted (Supplementary Table S2).

**Table 2:** Associations of triglyceride-glucose index, blood pressure, and hypertension with stroke.

Variables	No. of event/total	Model 1^a^		Model 2^b^		Model 3^c^	
		HR (95% CI)	*P* value	HR (95% CI)	*P* value	HR (95% CI)	*P* value
TyG index							
Quartiles							
Q1 (5.18, 8.23)	100/2,288	Reference		Reference		Reference	
Q2 (8.23, 8.60)	139/2,285	1.38 (1.07–1.78)	0.013	1.33 (1.03–1.72)	0.028	1.27 (0.98–1.65)	0.072
Q3 (8.60, 9.05)	192/2,286	1.93 (1.52–2.46)	<0.001	1.74 (1.36–2.22)	<0.001	1.55 (1.19–2.02)	0.001
Q4 (9.05, 13.00)	183/2,286	1.86 (1.46–2.37)	<0.001	1.60 (1.25–2.06)	<0.001	1.21 (0.88–1.66)	0.251
Per-SD increase	614/9,145	1.25 (1.17–1.34)	<0.001	1.19 (1.11–1.28)	<0.001	1.16 (1.02–1.33)	0.026
Hypertension							
No	222/5,388	Reference		Reference		Reference	
Yes	392/3,757	2.50 (2.11–2.96)	<0.001	2.28 (1.92–2.72)	<0.001	2.10 (1.76–2.50)	<0.001
Systole blood pressure, mmHg							
Quartiles							
Q1 (60.5, 114)	101/2,377	Reference		Reference		Reference	
Q2 (114, 126)	115/2,236	1.21 (0.93–1.57)	0.163	1.16 (0.89–1.51)	0.284	1.13 (0.87–1.47)	0.365
Q3 (126, 142)	162/2,253	1.69 (1.32–2.17)	<0.001	1.56 (1.21–2.01)	0.001	1.55 (1.20–1.99)	0.001
Q4 (142, 230)	236/2,279	2.41 (1.90–3.06)	<0.001	2.15 (1.68–2.76)	<0.001	2.05 (1.60–2.62)	<0.001
Per-SD increase	614/9,145	1.40 (1.30–1.50)	<0.001	1.36 (1.26–1.46)	<0.001	1.33 (1.24–1.43)	<0.001
Blood pressure status							
<140 mmHg	361/6,626	Reference		Reference		Reference	
≥140 mmHg	253/2,519	1.81 (1.54–2.14)	<0.001	1.68 (1.42–1.99)	<0.001	1.62 (1.37–1.92)	<0.001
Diastolic blood pressure, mmHg							
Quartiles							
Q1 (33.5, 67)	109/2,409	Reference		Reference		Reference	
Q2 (67, 74.5)	126/2,264	1.25 (0.97–1.61)	0.082	1.20 (0.93–1.55)	0.154	1.19 (0.92–1.53)	0.193
Q3 (74.5, 83)	153/2,257	1.56 (1.22–1.98)	<0.001	1.46 (1.14–1.86)	0.003	1.44 (1.13–1.85)	0.004
Q4 (83, 142)	226/2,215	2.46 (1.97–3.09)	<0.001	2.19 (1.74–2.76)	<0.001	2.11 (1.68–2.66)	<0.001
Per-SD increase	614/9,145	1.39 (1.30–1.49)	<0.001	1.34 (1.25–1.44)	<0.001	1.32 (1.23–1.42)	<0.001
Blood pressure status							
<90 mmHg	494/8,058	Reference		Reference		Reference	
≥90 mmHg	120/1,087	1.96 (1.61–2.38)	<0.001	1.76 (1.44–2.15)	<0.001	1.70 (1.39–2.08)	<0.001

CI, confidence interval; HR, hazard ratio; SD, standard deviation; TyG, triglyceride-glucose.

^a^ Adjusted for age, gender.

^b^ Adjusted for age, gender, marital status, residence, education level, body mass index, smoking status, and drinking status.

^c^ Adjusted as model 2 plus diabetes, heart disease, dyslipidemia, kidney disease, history of medication use for diabetes, history of medication use for dyslipidemia, TC, HDL-C, LDL-C, HbA1c, hsCRP, and eGFR.

### 3.3 Association of TyG index with hypertension

Table 3 shows the associations between TyG index and incident hypertension. After adjusting for confounders (in model 3), by comparing Quartile 4 with Quartile 1, the adjusted ORs for incident hypertension were 1.52 (95% CI, 1.27–1.81). A one SD increase in TyG index was associated with an elevated risk of hypertension (OR, 1.29; 95% CI, 1.19–1.41). A linear and positive association between the TyG index and risk of incident hypertension using RCS regression was also found (for association, *P* < 0.001; for nonlinear, *P* = 0.438; Supplementary Figure S5D). Similar results were found when complete data analyses were conducted (Supplementary Table S3).

**Table 3:** Association of triglyceride-glucose index with hypertension.

TyG index	No. of event/total	Model 1^a^		Model 2^b^		Model 3^c^	
		OR (95% CI)	*P* value	OR (95% CI)	*P* value	OR (95% CI)	*P* value
Quartiles							
Q1 (5.18, 8.23)	722/2,288	Reference		Reference		Reference	
Q2 (8.23, 8.60)	818/2,285	1.20 (1.06–1.36)	0.004	1.11 (0.98–1.27)	0.098	1.07 (0.94–1.22)	0.305
Q3 (8.60, 9.05)	1,022/2,286	1.75 (1.55–1.98)	<0.001	1.47 (1.30–1.67)	<0.001	1.37 (1.19–1.58)	<0.001
Q4 (9.05, 13.00)	1,195/2,286	2.45 (2.17–2.78)	<0.001	1.86 (1.63–2.12)	<0.001	1.52 (1.27–1.81)	<0.001
Per-SD increase	3,757/9,145	1.44 (1.37–1.50)	<0.001	1.29 (1.24–1.36)	<0.001	1.29 (1.19–1.41)	<0.001

CI, confidence interval; OR, odds ratio; SD, standard deviation; TyG, triglyceride-glucose.

^a^ Adjusted for age, gender.

^b^ Adjusted for age, gender, marital status, residence, education level, body mass index, smoking status, and drinking status.

^c^ Adjusted as model 2 plus diabetes, heart disease, dyslipidemia, kidney disease, history of medication use for diabetes, history of medication use for dyslipidemia, TC, HDL-C, LDL-C, HbA1c, hsCRP, and eGFR.

### 3.4 Interaction and joint analysis

**Figure 1:**
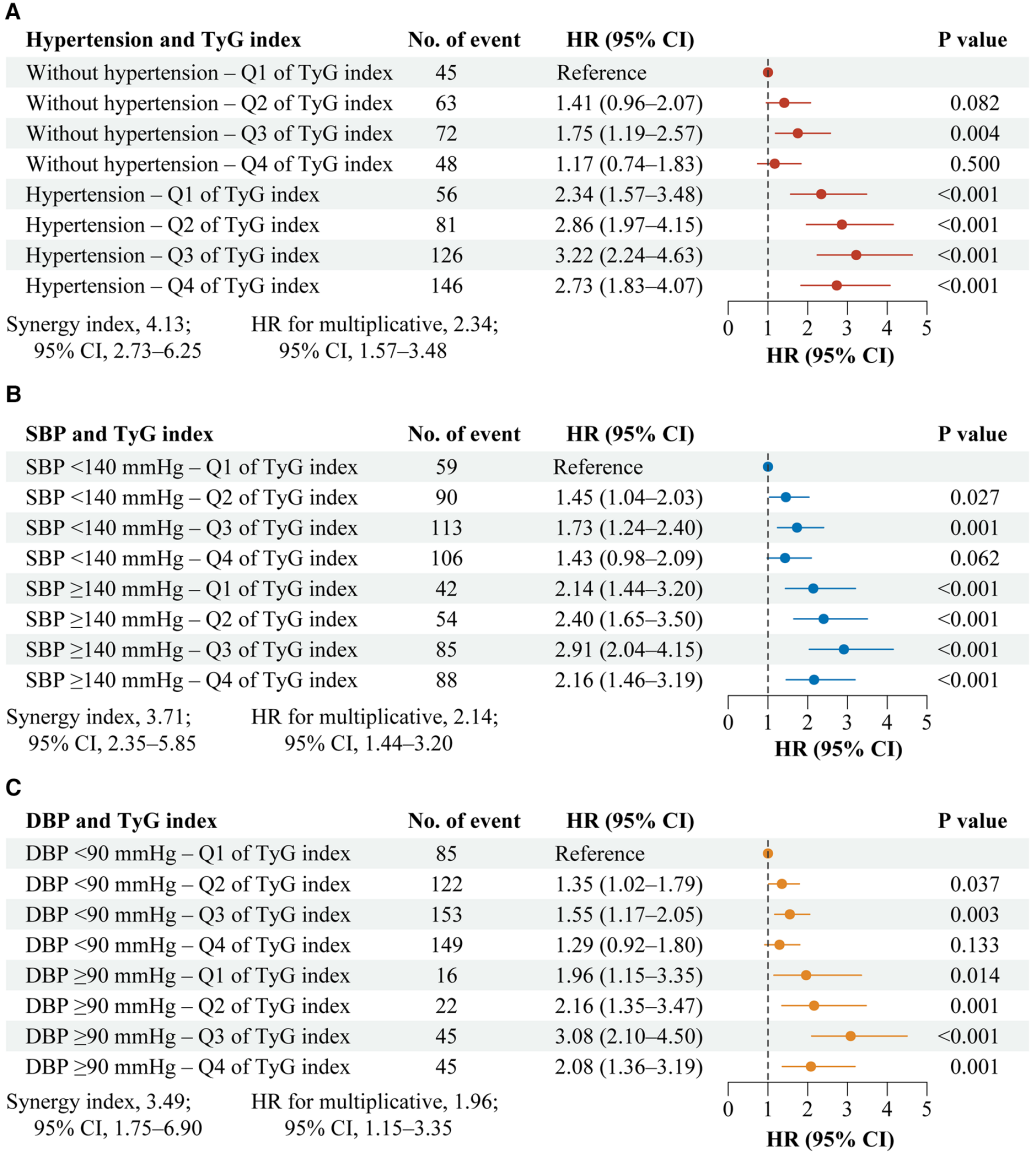
Interaction and joint effects for exposures to TyG index and hypertension on stroke. Graphs show the interaction and joint effects of exposure to the TyG index and hypertension (**A**), systolic blood pressure (**B**), and diastolic blood pressure (**C**) in relation to stroke. All models were adjusted for age, gender, marital status, residence, education level, body mass index, smoking status, drinking status, diabetes, heart disease, dyslipidemia, kidney disease, history of medication use for diabetes, history of medication use for dyslipidemia, TC, HDL-C, LDL-C, HbA1c, hsCRP, and eGFR. Additive interaction was evaluated using SI between the TyG index and hypertension, and the additive interaction was statistically significant when its CIs did not include 1. Multiplicative interaction was evaluated using HRs for the product term between the TyG index and hypertension, and the multiplicative interaction was statistically significant when its CIs did not include 1. CI, confidence interval; DBP, diastolic blood pressure; HR, hazard ratio; SBP, systole blood pressure; TyG, triglyceride-glucose.

Figure 1 shows the interaction and joint association of TyG index and hypertension on the stroke. Both multiplicative and additive interactions were observed between TyG index and hypertension on stroke (HR for multiplicative: 2.34, 95% CI, 1.57–3.48; SI: 4.13, 95% CI, 2.73–6.25; Figure 1A). Similar patterns were found for TyG index and systole blood pressure on stroke (Figure 1B), as well as, TyG index and diastolic blood pressure on stroke (Figure 1C). The results remained similar in all sensitivity analyses (Supplementary Table S4) and subgroup analyses (Supplementary Tables S5–S6).

### 3.5 Mediation analyses

**Figure 2:**
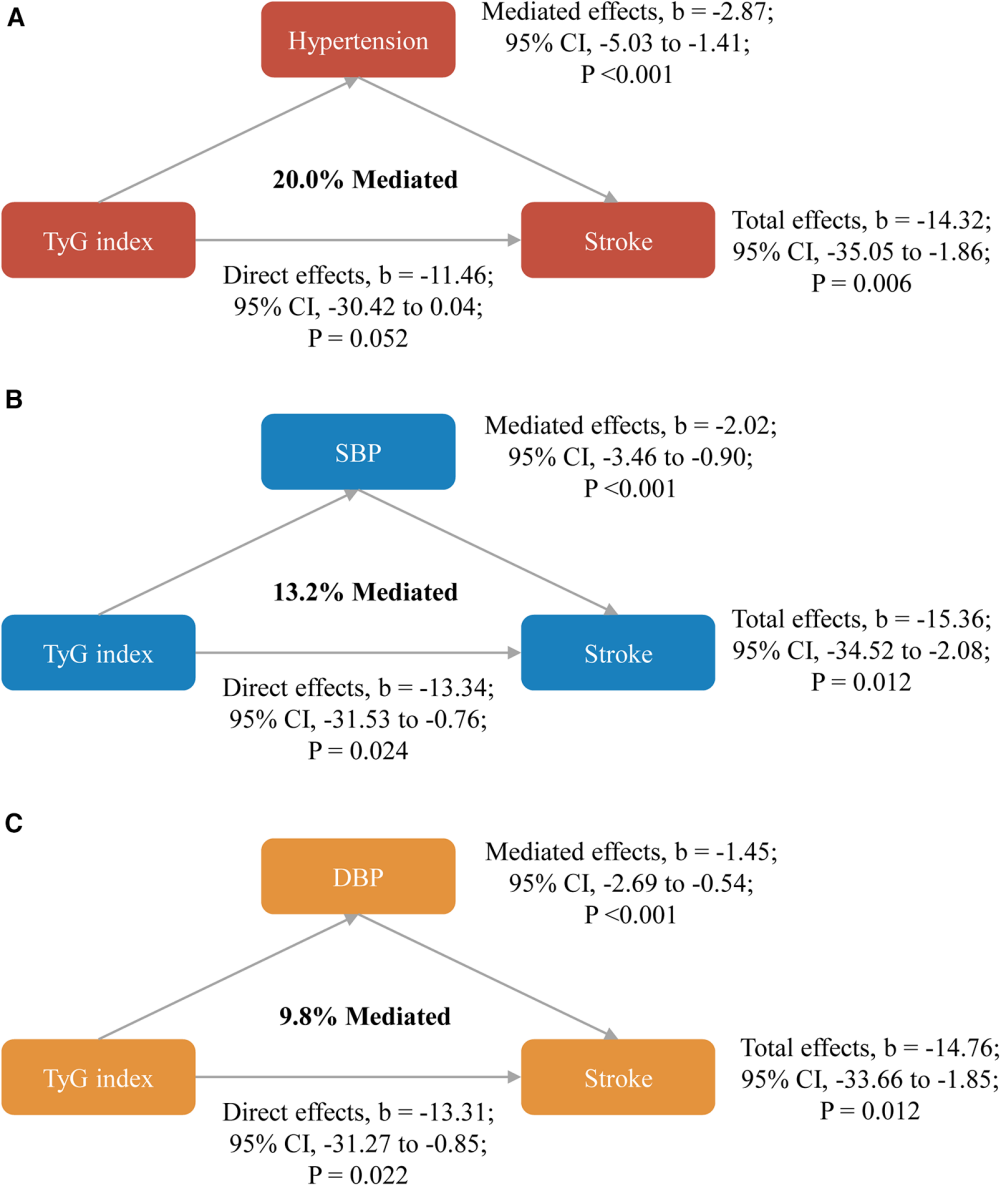
Mediated effects by hypertension (**A**), systole blood pressure (**B**) or diastolic blood pressure (**C**) on the associations of TyG index with stroke. All models were adjusted for age, gender, marital status, residence, education level, body mass index, smoking status, drinking status, diabetes, heart disease, dyslipidemia, kidney disease, history of medication use for diabetes, history of medication use for dyslipidemia, TC, HDL-C, LDL-C, HbA1c, hsCRP, and eGFR. CI, confidence interval; DBP, diastolic blood pressure; SBP, systole blood pressure; TyG, triglyceride-glucose.

As shown in Figure 2, significant mediated effects by hypertension, systole blood pressure, or diastolic blood pressure were observed on the associations between TyG index and stroke, the mediated proportion were 20.0%, 13.2%, and 9.8%, respectively. Similar results were found when complete data analyses were conducted (Supplementary Table S7), used blood pressure data at Wave 2 (Supplementary Table S8), or stratified by gender (Supplementary Table S9**)**. However, the mediated proportion was more pronounced among participants with age <60 years when compared with those with age ≥60 years (mediated by hypertension: 27.6% vs. 14.3%; by systole blood pressure: 24.6% vs. 6.9%; by diastolic blood pressure: 20.4% vs. 4.1%; Supplementary Table S10).

## 4 Discussion

Our findings affirm a synergistic effect between the TyG index and hypertension in elevating stroke risk in middle-aged and older Chinese adults. Interestingly, a modest portion of the relationship between the TyG index and stroke was mediated by hypertension. This insight indicates that a coordinated approach to controlling both risk factors could provide additional benefits in stroke prevention.

The findings of this study provide substantial evidence for the presence of a synergistic effect between the TyG index and hypertension on the risk of stroke. Both TyG index and hypertension are established risk factors for cardiovascular diseases (12, 13, 25, 26), and their association with stroke is well-documented (4, 8, 27, 28). However, this study goes a step further by demonstrating that when both factors coexist, their combined impact on stroke risk is greater than the sum of their individual effects. This implies that patients with high TyG index and hypertension are at a disproportionately higher risk of suffering from a stroke. Multiple pathways may contribute to this synergy, including their shared role in promoting endothelial dysfunction, inflammation, oxidative stress, and atherosclerosis. Hyperglycemia and insulin resistance, reflected in the TyG index, are known to impair endothelial function and promote inflammation (29–32). In parallel, hypertension exerts mechanical stress on the arterial walls, leading to endothelial dysfunction and inflammation (33, 34). Additionally, insulin resistance has been linked to increased sympathetic nervous system activity, further exacerbating hypertension (35, 36). It is essential to acknowledge that the mechanisms underlying this synergistic effect might be more complex than a simple additive interaction. The synergistic effect could involve synergistic mechanisms, where the presence of both factors leads to a non-linear, amplified increase in stroke risk. Alternatively, the interaction might be multiplicative, implying that the combined effect is proportional to the product of the individual risks. Further research is warranted to delve into the precise nature of this interaction and its clinical implications.

The mediation analysis in this study revealed that hypertension partially mediated the association between the TyG index and stroke. In other words, while the TyG index has a direct impact on stroke risk, a portion of this effect is channeled through its influence on hypertension. This finding underscores the importance of controlling hypertension in individuals with elevated TyG index to mitigate their stroke risk. One possible explanation for this mediation effect is the role of insulin resistance in the development of hypertension. Insulin resistance is a central component of the TyG index, as it reflects the interaction between triglycerides and glucose (10, 11). Insulin resistance is associated with multiple metabolic abnormalities, including impaired glucose metabolism and dyslipidemia, all of which contribute to an increased risk of hypertension (37, 38). Furthermore, insulin resistance is linked to endothelial dysfunction, oxidative stress, and inflammation, which can lead to hypertension (29–32, 35). Another possible explanation is the effect of the TyG index on the endothelium. Endothelial dysfunction plays a pivotal role in the pathogenesis of hypertension and atherosclerosis (34). Studies have demonstrated that insulin resistance is associated with impaired endothelial function (29, 30), which may contribute to hypertension by reducing the ability of blood vessels to relax (34). Therefore, the TyG index, as a marker of insulin resistance, may indirectly affect blood pressure by influencing endothelial function.

The identification of the synergistic effect between TyG index and hypertension and the mediation role of hypertension in the association between TyG index and stroke has significant implications for stroke prevention and management. First, prevention Strategies Primary prevention efforts should focus on reducing the risk factors associated with both TyG index and hypertension. Lifestyle modifications, such as dietary improvements, regular physical activity, and weight management, have proven to be effective strategies for addressing both conditions (39–41). These lifestyle changes can improve insulin sensitivity, reduce blood pressure, and ultimately lower the risk of stroke. Second, Screening and Risk Assessment Healthcare providers should consider assessing the TyG index in addition to traditional risk factors like blood pressure and lipid profiles when evaluating stroke risk in patients. A high TyG index may warrant closer monitoring and more aggressive management of hypertension. Incorporating the TyG index into existing risk assessment tools can lead to more precise risk stratification, enabling healthcare professionals to identify high-risk patients who may benefit from early interventions (5, 15). Third, the synergistic effect observed in this study highlights the need for individualized treatment plans. Patients with high TyG index and hypertension may benefit from more intensive management of both conditions, potentially including pharmacological interventions alongside lifestyle modifications. Healthcare providers should work closely with patients to develop personalized treatment plans that address their specific risk factors. Treatment strategies can include antihypertensive medications, statins to manage dyslipidemia, and diabetes management for those with glucose abnormalities.

The strengths of this study included the prospective design and the interactive effects of TyG index and hypertension on stroke. However, several limitations need to be addressed. First, we excluded participants who did not have blood samples, which could lead to selective bias. Second, some confounding factors of the association between TyG index and stroke, such as diet (42), physical activity (43), and family history of stroke (44) were not adjusted in this study. Third, as with many studies, stroke diagnosis in this research was based on self-reporting, posing a methodological challenge. The CHARLS dataset lacks medical records, making it impossible to validate self-reported stroke incidents. Nonetheless, it's pertinent to highlight that other large-scale studies, like the English Longitudinal Study of Ageing, have found a commendable concordance between self-reported strokes and medical documentation (45). Fourth, the BMI and the TyG were measured concurrently, raising the possibility of reverse causality. However, this concern was addressed through sensitivity analyses using hypertension data from Wave 2, which yielded comparable results. Five, only participants from China were involved in this study; thus the findings may not fully generalize to other countries. In addition, time-varying exposures were not included in the present analysis, so residual confounding is a concern.

## 5 Conclusions

This study provides compelling evidence of the synergistic effect between the TyG index and hypertension on stroke risk in middle-aged and older Chinese adults. Hypertension mediates a portion of the association between TyG index and stroke, emphasizing the importance of coordinated control strategies for both risk factors. These findings have significant implications for stroke prevention and management, suggesting that addressing TyG index and hypertension together may be more effective in reducing the risk of stroke. Further research is needed to fully understand the mechanisms underlying this interaction and to develop tailored interventions for high-risk individuals. Public health initiatives and clinical practice guidelines should take these findings into account to improve stroke prevention strategies.

## Data Availability

Publicly available datasets were analyzed in this study. This data can be found here: http://charls.pku.edu.cn/en.
