# Supplementary material for: Interacting and joint effects of triglyceride-glucose index and hypertension on stroke risk in middle-aged and older Chinese adults: a population-based prospective cohort study
Source: Front Cardiovasc Med. 2024 May 15;11:1363049. doi: 10.3389/fcvm.2024.1363049 (PMC11133867; doi:10.3389/fcvm.2024.1363049)
Supplement: Supplementary file 1 [file Datasheet1.docx]

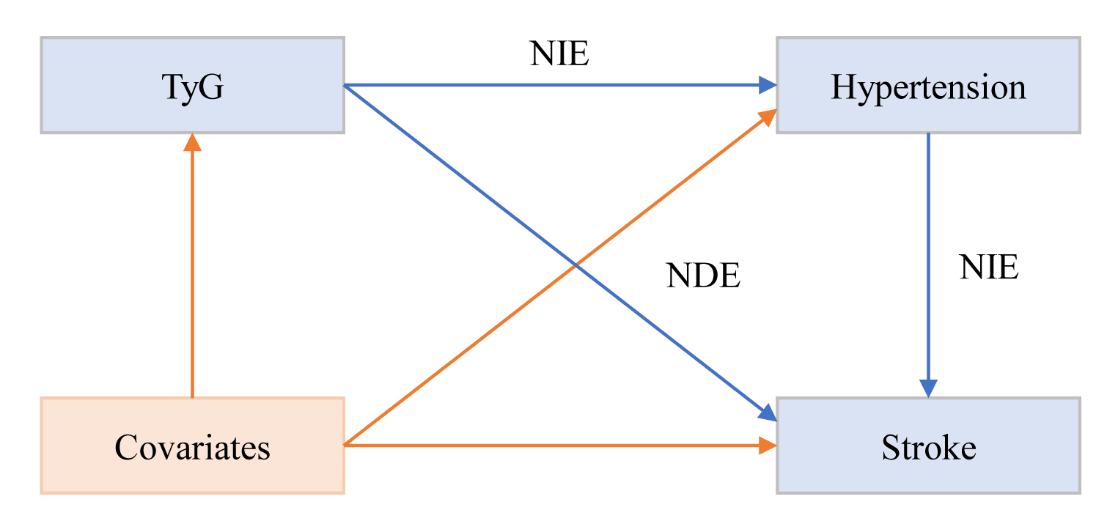


**Figure S1: Mediating pathway of the association of TyG index with stroke.**

Direct acyclic graph of a structural model of mediation of the association between TyG index and stroke by hypertension. **Abbreviations:** TyG, triglyceride-glucose; NDE, natural direct effects; NIE, natural indirect effects.


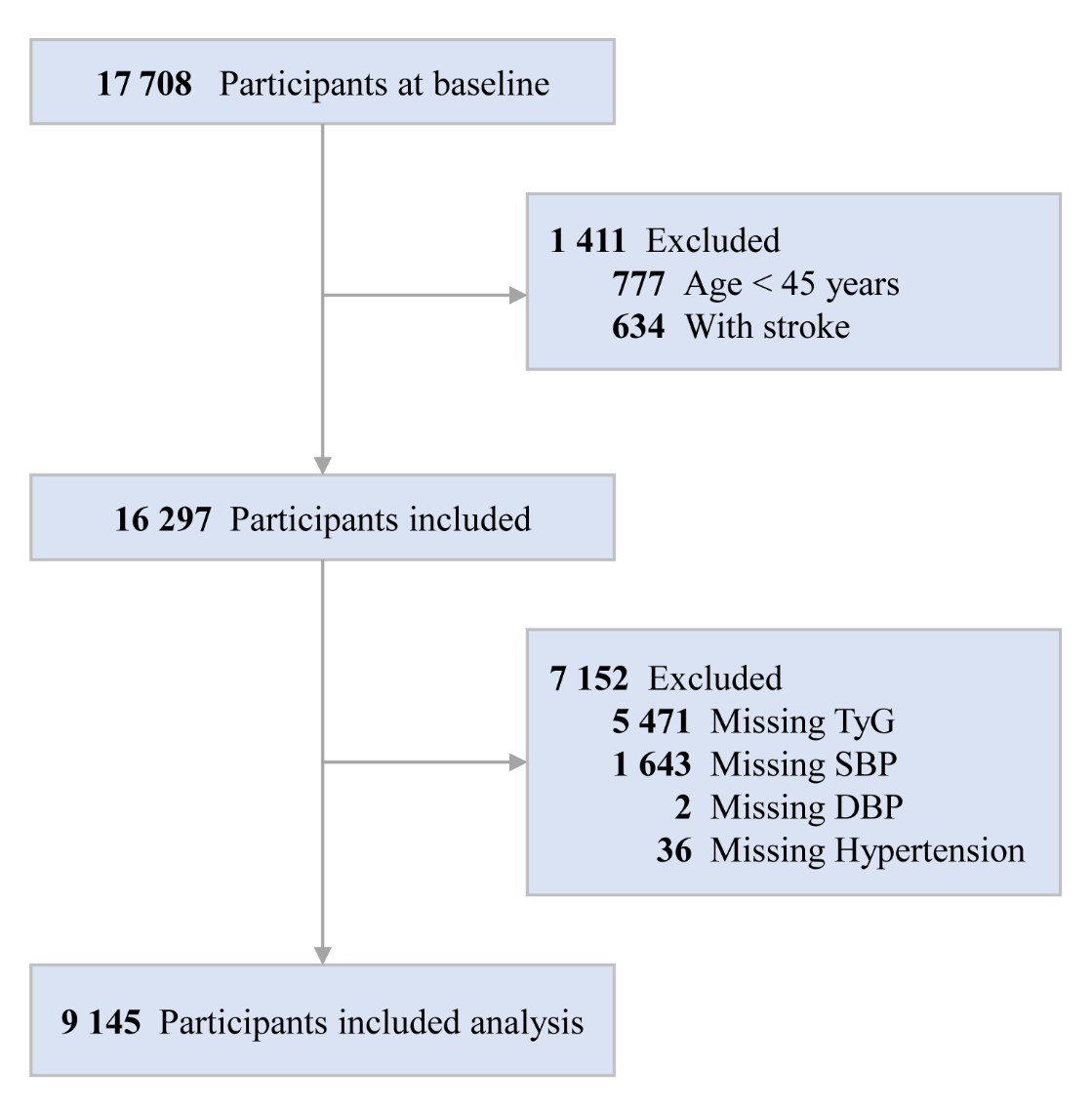


**Figure S2: Flowchart of the study population.**

**Abbreviations**: DBP, diastolic blood pressure; SBP, systole blood pressure; TyG, triglyceride-glucose.


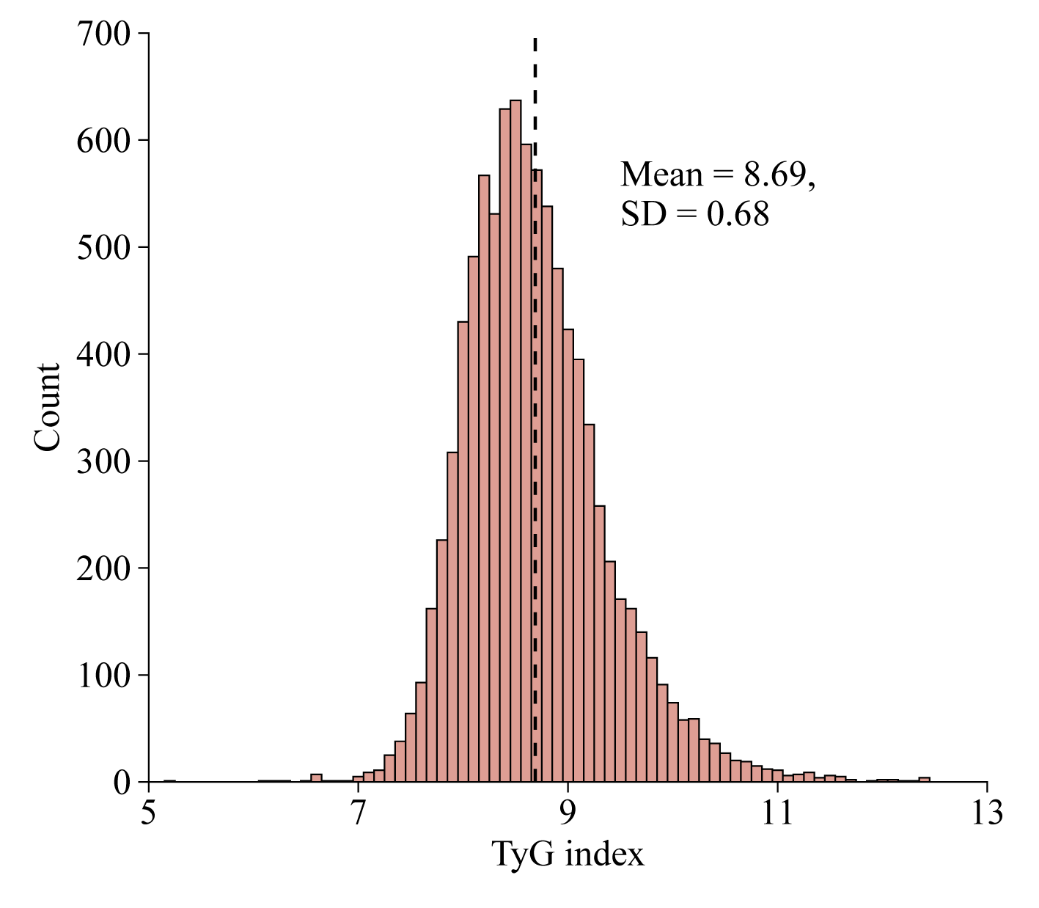


**Figure S3: Distribution for triglyceride-glucose index.**


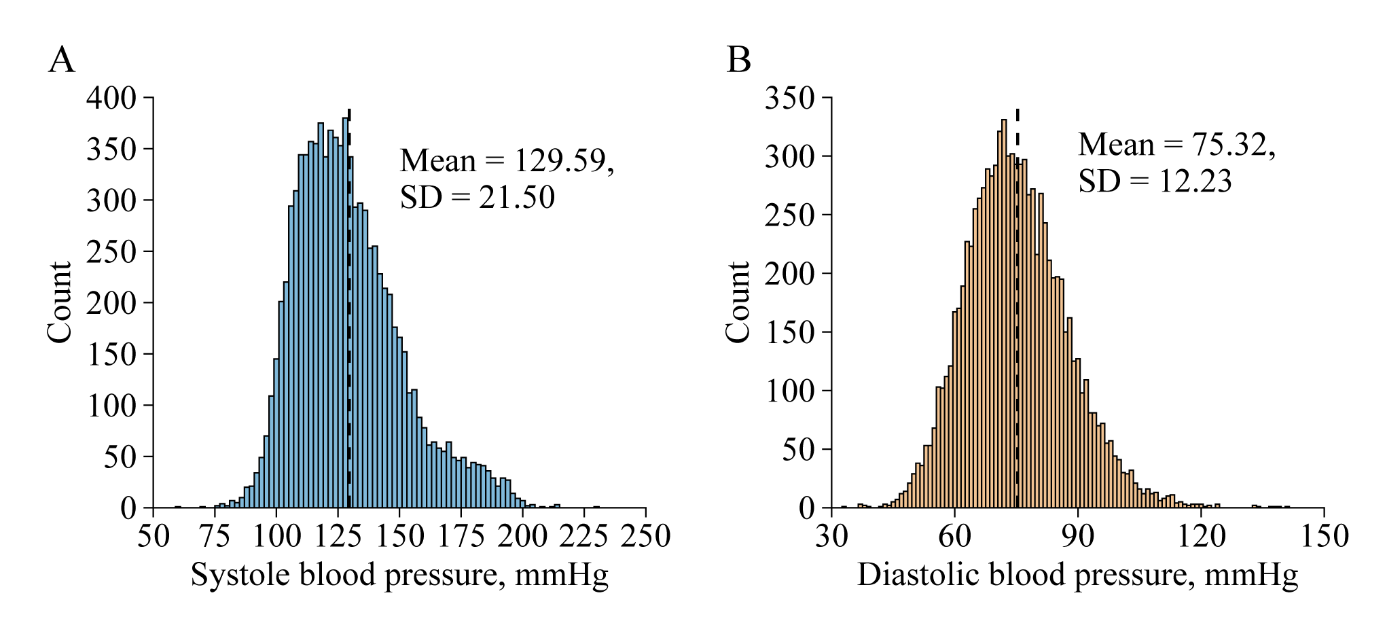


**Figure S4: Distribution for systole blood pressure (A) and diastolic blood pressure (B).**

**
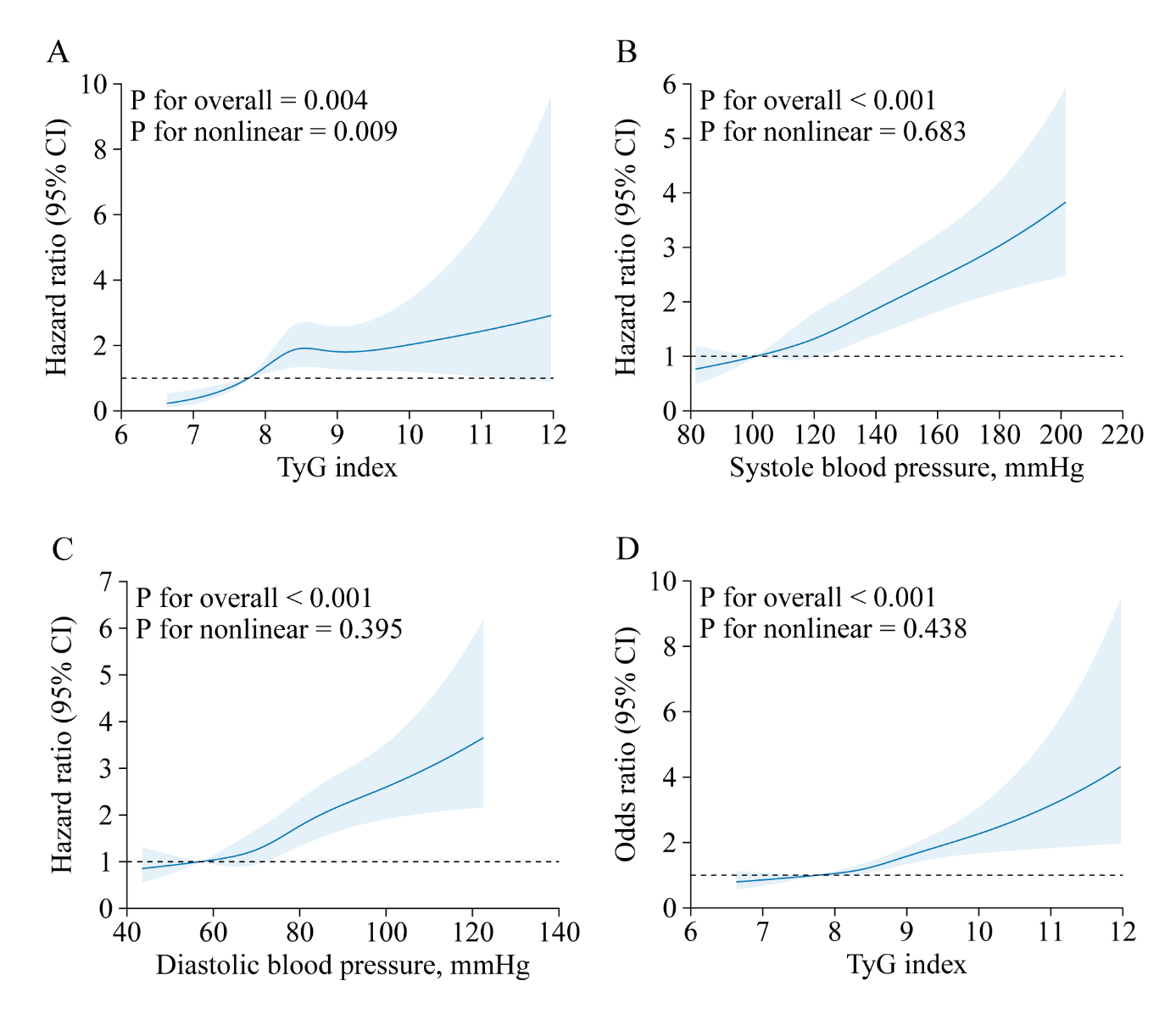
**

**Figure S5: Associations of triglyceride-glucose (TyG) index, blood pressure with stroke, and association between triglyceride-glucose index and hypertension.**

Graphs show hazard ratios (HRs) for stroke according to TyG index (**A**) systole blood pressure (**B**), and diastolic blood pressure (**C**) using restricted cubic spline Cox proportional hazards regression models; and odds ratios (ORs) for hypertension according to TyG index (**D**) using restricted cubic spline logistic regression model. All models were adjusted for age, gender, marital status, residence, education level, body mass index, smoking status, drinking status, diabetes, heart disease, dyslipidemia, kidney disease, history of medication use for diabetes, history of medication use for dyslipidemia, TC, HDL-C, LDL-C, HbA1c, hsCRP, and eGFR. Solid lines indicate HRs or ORs, while shaded areas denote the corresponding 95% confidence intervals (CIs).

Table S1: Baseline characteristics between participants included and not included

| Characteristic | Exclude (n=8563) | Include (n=9145) | P value ^a^ |
| --- | --- | --- | --- |
| Age, years |  |  | <0.001 |
| <60 | 5147 (60.1%) | 5096 (55.7%) |  |
| ≥60 | 3241 (37.8%) | 4049 (44.3%) |  |
| Missing | 175 (2.0%) | 0 (0.0%) |  |
| Gender |  |  | <0.001 |
| Male | 4227 (49.4%) | 4251 (46.5%) |  |
| Female | 4334 (50.6%) | 4894 (53.5%) |  |
| Missing | 2 (0.0%) | 0 (0.0%) |  |
| Marital status |  |  | <0.001 |
| Married | 6538 (76.4%) | 7632 (83.5%) |  |
| Other | 2013 (23.5%) | 1513 (16.5%) |  |
| Missing | 12 (0.1%) | 0 (0.0%) |  |
| Residence |  |  | <0.001 |
| Urban | 3972 (46.4%) | 3199 (35.0%) |  |
| Rural | 4591 (53.6%) | 5946 (65.0%) |  |
| Education level |  |  | <0.001 |
| No formal education | 2131 (24.9%) | 2720 (29.7%) |  |
| Primary school | 3195 (37.3%) | 3725 (40.7%) |  |
| Middle or high school | 2678 (31.3%) | 2425 (26.5%) |  |
| College or above | 543 (6.3%) | 275 (3.0%) |  |
| Missing | 16 (0.2%) | 0 (0.0%) |  |
| Body mass index, kg/m^2^ |  |  | 0.573 |
| <18.5 | 324 (3.8%) | 625 (6.8%) |  |
| 18.5-23.9 | 2464 (28.8%) | 4730 (51.7%) |  |
| 24.0-27.9 | 1303 (15.2%) | 2639 (28.9%) |  |
| ≥28.0 | 512 (6.0%) | 1034 (11.3%) |  |
| Missing | 3960 (46.2%) | 117 (1.3%) |  |
| Smoking status |  |  | <0.001 |
| Never | 5067 (59.2%) | 5552 (60.7%) |  |
| Former | 623 (7.3%) | 794 (8.7%) |  |
| Current | 2099 (24.5%) | 2772 (30.3%) |  |
| Missing | 774 (9.0%) | 27 (0.3%) |  |
| Drinking status |  |  | 0.952 |
| Never | 4940 (57.7%) | 5393 (59.0%) |  |
| Former | 691 (8.1%) | 752 (8.2%) |  |
| Current | 2772 (32.4%) | 2995 (32.8%) |  |
| Missing | 160 (1.9%) | 5 (0.1%) |  |
| Diabetes |  |  | 0.909 |
| No | 7833 (91.5%) | 8513 (93.1%) |  |
| Yes | 507 (5.9%) | 555 (6.1%) |  |
| Missing | 223 (2.6%) | 77 (0.8%) |  |
| Heart disease |  |  | 0.080 |
| No | 7294 (85.2%) | 8043 (87.9%) |  |
| Yes | 1056 (12.3%) | 1074 (11.7%) |  |
| Missing | 213 (2.5%) | 28 (0.3%) |  |
| Dyslipidemia |  |  | 0.252 |
| No | 7394 (86.3%) | 8123 (88.8%) |  |
| Yes | 833 (9.7%) | 863 (9.4%) |  |
| Missing | 336 (3.9%) | 159 (1.7%) |  |
| Kidney disease |  |  | 0.055 |
| No | 7907 (92.3%) | 8566 (93.7%) |  |
| Yes | 439 (5.1%) | 540 (5.9%) |  |
| Missing | 217 (2.5%) | 39 (0.4%) |  |
| History of medication use for diabetes |  |  | 0.127 |
| No | 7976 (93.1%) | 8716 (95.3%) |  |
| Yes | 361 (4.2%) | 351 (3.8%) |  |
| Missing | 226 (2.6%) | 78 (0.9%) |  |
| History of medication use for dyslipidemia |  |  | 0.743 |
| No | 7801 (91.1%) | 8535 (93.3%) |  |
| Yes | 418 (4.9%) | 447 (4.9%) |  |
| Missing | 344 (4.0%) | 163 (1.8%) |  |
| Systole blood pressure, mmHg |  |  | 0.875 |
| Median (IQR) | 126.00 (114.00, 142.00) | 126.50 (114.00, 141.50) |  |
| Missing | 3968 | 0 |  |
| Diastolic blood pressure, mmHg |  |  | 0.012 |
| Median (IQR) | 75.00 (67.50, 84.00) | 74.50 (67.00, 83.00) |  |
| Missing | 3969 | 0 |  |

^a^ P value was based on χ^2^ or Kruskal-Wallis rank sum test where appropriate

Table S2: Associations of triglyceride-glucose index, blood pressure, and hypertension with stroke in subpopulations of 8, 693 participants with complete data

| Variables | No. of event / total | Model 1 ^a^ | |  | Model 2 ^b^ | |  | Model 3 ^c^ | |
| --- | --- | --- | --- | --- | --- | --- | --- | --- | --- |
|  |  | HR (95% CI) | P value |  | HR (95% CI) | P value |  | HR (95% CI) | P value |
| TyG index |  |  |  |  |  |  |  |  |  |
| Quartiles |  |  |  |  |  |  |  |  |  |
| Q1 [5.18, 8.23] | 95 / 2188 | Reference |  |  | Reference |  |  | Reference |  |
| Q2 (8.23, 8.60] | 141 / 2185 | 1.49 (1.15–1.93) | 0.003 |  | 1.43 (1.10–1.85) | 0.008 |  | 1.33 (1.02–1.74) | 0.034 |
| Q3 (8.60, 9.05] | 190 / 2160 | 2.06 (1.61–2.64) | <0.001 |  | 1.84 (1.43–2.36) | <0.001 |  | 1.57 (1.20–2.06) | 0.001 |
| Q4 (9.05, 13.00] | 184 / 2160 | 2.03 (1.58–2.60) | <0.001 |  | 1.72 (1.33–2.22) | <0.001 |  | 1.21 (0.87–1.68) | 0.251 |
| Per-SD increase | 610 / 8693 | 1.29 (1.20–1.39) | <0.001 |  | 1.23 (1.13–1.32) | <0.001 |  | 1.18 (1.02–1.36) | 0.027 |
| Hypertension |  |  |  |  |  |  |  |  |  |
| No | 221 / 5144 | Reference |  |  | Reference |  |  | Reference |  |
| Yes | 389 / 3549 | 2.51 (2.12–2.97) | <0.001 |  | 2.27 (1.91–2.70) | <0.001 |  | 2.09 (1.75–2.49) | <0.001 |
| Systole blood pressure, mmHg |  |  |  |  |  |  |  |  |  |
| Quartiles |  |  |  |  |  |  |  |  |  |
| Q1 [60.5, 114] | 99 / 2276 | Reference |  |  | Reference |  |  | Reference |  |
| Q2 (114, 126] | 111 / 2122 | 1.19 (0.91–1.57) | 0.199 |  | 1.14 (0.87–1.49) | 0.357 |  | 1.11 (0.85–1.46) | 0.449 |
| Q3 (126, 142] | 156 / 2130 | 1.67 (1.29–2.15) | <0.001 |  | 1.51 (1.17–1.96) | 0.002 |  | 1.50 (1.16–1.94) | 0.002 |
| Q4 (142, 230] | 244 / 2165 | 2.54 (2.01–3.23) | <0.001 |  | 2.24 (1.76–2.86) | <0.001 |  | 2.12 (1.66–2.71) | <0.001 |
| Per-SD increase | 610 / 8693 | 1.44 (1.34–1.54) | <0.001 |  | 1.39 (1.29–1.49) | <0.001 |  | 1.36 (1.26–1.47) | <0.001 |
| Blood pressure status |  |  |  |  |  |  |  |  |  |
| <140 mmHg | 349 / 6306 | Reference |  |  | Reference |  |  | Reference |  |
| ≥140 mmHg | 261 / 2387 | 1.94 (1.64–2.28) | <0.001 |  | 1.79 (1.51–2.11) | <0.001 |  | 1.72 (1.46–2.04) | <0.001 |
| Diastolic blood pressure, mmHg |  |  |  |  |  |  |  |  |  |
| Quartiles |  |  |  |  |  |  |  |  |  |
| Q1 [33.5,67] | 110 / 2301 | Reference |  |  | Reference |  |  | Reference |  |
| Q2 (67, 74.5] | 126 / 2159 | 1.24 (0.96–1.61) | 0.094 |  | 1.18 (0.92–1.53) | 0.195 |  | 1.16 (0.90–1.50) | 0.259 |
| Q3 (74.5, 83] | 146 / 2136 | 1.48 (1.16–1.90) | 0.002 |  | 1.36 (1.06–1.75) | 0.015 |  | 1.34 (1.04–1.72) | 0.022 |
| Q4 (83, 142] | 228 / 2097 | 2.50 (1.99–3.14) | <0.001 |  | 2.20 (1.75–2.78) | <0.001 |  | 2.11 (1.67–2.67) | <0.001 |
| Per-SD increase | 610 / 8693 | 1.41 (1.31–1.51) | <0.001 |  | 1.35 (1.26–1.46) | <0.001 |  | 1.33 (1.24–1.44) | <0.001 |
| Blood pressure status |  |  |  |  |  |  |  |  |  |
| <90 mmHg | 487 / 7667 | Reference |  |  | Reference |  |  | Reference |  |
| ≥90 mmHg | 123 / 1026 | 2.10 (1.72–2.55) | <0.001 |  | 1.89 (1.55–2.32) | <0.001 |  | 1.84 (1.50–2.25) | <0.001 |

Abbreviations: CI, confidence interval; HR, hazard ratio; SD, standard deviation; TyG, triglyceride-glucose.

^a^ Adjusted for age, and gender.

^b^ Adjusted for age, gender, marital status, residence, education level, body mass index, smoking status, and drinking status.

^c^ Adjusted as model 2 plus diabetes, heart disease, dyslipidemia, kidney disease, history of medication use for diabetes, history of medication use for dyslipidemia, TC, HDL-C, LDL-C, HbA1c, hsCRP, and eGFR.

Table S3: Association of triglyceride-glucose index with hypertension in subpopulations of 8,693 participants with complete data

| TyG index | No. of event / total | Model 1 ^a^ | |  | Model 2 ^b^ | |  | Model 3 ^c^ | |
| --- | --- | --- | --- | --- | --- | --- | --- | --- | --- |
|  |  | OR (95% CI) | P value |  | OR (95% CI) | P value |  | OR (95% CI) | P value |
| Quartiles |  |  |  |  |  |  |  |  |  |
| Q1 [5.18, 8.23] | 685 / 2188 | Reference |  |  | Reference |  |  | Reference |  |
| Q2 (8.23, 8.60] | 783 / 2185 | 1.21 (1.07–1.38) | 0.003 |  | 1.12 (0.98–1.28) | 0.083 |  | 1.09 (0.95–1.24) | 0.231 |
| Q3 (8.60, 9.05] | 963 / 2160 | 1.77 (1.56–2.00) | <0.001 |  | 1.48 (1.30–1.69) | <0.001 |  | 1.39 (1.21–1.61) | <0.001 |
| Q4 (9.05, 13.00] | 1118 / 2160 | 2.43 (2.14–2.75) | <0.001 |  | 1.83 (1.60–2.09) | <0.001 |  | 1.51 (1.26–1.81) | <0.001 |
| Per-SD increase | 3549 / 8693 | 1.43 (1.36–1.49) | <0.001 |  | 1.28 (1.22–1.35) | <0.001 |  | 1.27 (1.17–1.39) | <0.001 |

Abbreviations: CI, confidence interval; OR, odds ratio; SD, standard deviation; TyG, triglyceride-glucose.

^a^ Adjusted for age, and gender.

^b^ Adjusted for age, gender, marital status, residence, education level, body mass index, smoking status, and drinking status.

^c^ Adjusted as model 2 plus diabetes, heart disease, dyslipidemia, kidney disease, history of medication use for diabetes, history of medication use for dyslipidemia, TC, HDL-C, LDL-C, HbA1c, hsCRP, and eGFR.

Table S4: Interaction and joint effects for exposures to TyG index and hypertension on stroke in subpopulations of 8,693 participants with complete data ^a^

| Variable | No. of event | HR (95% CI) | P value |
| --- | --- | --- | --- |
| Hypertension and TyG index |  |  |  |
| Without hypertension – Q1 of TyG index | 43 | Reference |  |
| Without hypertension – Q2 of TyG index | 61 | 1.40 (0.95–2.08) | 0.091 |
| Without hypertension – Q3 of TyG index | 71 | 1.76 (1.19–2.60) | 0.005 |
| Without hypertension – Q4 of TyG index | 46 | 1.10 (0.70–1.75) | 0.677 |
| Hypertension – Q1 of TyG index | 52 | 2.32 (1.54–3.50) | <0.001 |
| Hypertension – Q2 of TyG index | 80 | 2.88 (1.97–4.21) | <0.001 |
| Hypertension – Q3 of TyG index | 119 | 3.10 (2.13–4.50) | <0.001 |
| Hypertension – Q4 of TyG index | 138 | 2.58 (1.71–3.90) | <0.001 |
| Synergy index | NA | 4.08 (2.64–6.29) | NA |
| HR for multiplicative | NA | 2.32 (1.54–3.50) | NA |
| SBP and TyG index |  |  |  |
| SBP <140 mmHg – Q1 of TyG index | 55 | Reference |  |
| SBP <140 mmHg – Q2 of TyG index | 88 | 1.49 (1.06–2.09) | 0.022 |
| SBP <140 mmHg – Q3 of TyG index | 107 | 1.69 (1.20–2.38) | 0.002 |
| SBP <140 mmHg – Q4 of TyG index | 99 | 1.36 (0.92–2.01) | 0.126 |
| SBP ≥140 mmHg – Q1 of TyG index | 40 | 2.20 (1.46–3.32) | <0.001 |
| SBP ≥140 mmHg – Q2 of TyG index | 53 | 2.45 (1.67–3.61) | <0.001 |
| SBP ≥140 mmHg – Q3 of TyG index | 83 | 3.00 (2.08–4.31) | <0.001 |
| SBP ≥140 mmHg – Q4 of TyG index | 85 | 2.09 (1.40–3.14) | <0.001 |
| Synergy index | NA | 3.86 (2.45–6.10) | NA |
| HR for multiplicative | NA | 2.20 (1.46–3.32) | NA |
| DBP and TyG index |  |  |  |
| DBP <90 mmHg – Q1 of TyG index | 80 | Reference |  |
| DBP <90 mmHg – Q2 of TyG index | 119 | 1.37 (1.02–1.82) | 0.034 |
| DBP <90 mmHg – Q3 of TyG index | 147 | 1.53 (1.14–2.04) | 0.005 |
| DBP <90 mmHg – Q4 of TyG index | 141 | 1.21 (0.86–1.72) | 0.271 |
| DBP ≥90 mmHg – Q1 of TyG index | 15 | 2.02 (1.16–3.51) | 0.013 |
| DBP ≥90 mmHg – Q2 of TyG index | 22 | 2.21 (1.37–3.55) | 0.001 |
| DBP ≥90 mmHg – Q3 of TyG index | 43 | 3.10 (2.10–4.59) | <0.001 |
| DBP ≥90 mmHg – Q4 of TyG index | 43 | 2.05 (1.32–3.17) | 0.001 |
| Synergy index | NA | 3.70 (1.80–7.61) | NA |
| HR for multiplicative | NA | 2.02 (1.16–3.51) | NA |

Abbreviations: CI, confidence interval; DBP, diastolic blood pressure; HR, hazard ratio; SBP, systole blood pressure; TyG, triglyceride-glucose.

^a^ All models were adjusted for age, gender, marital status, residence, education level, body mass index, smoking status, drinking status, diabetes, heart disease, dyslipidemia, kidney disease, history of medication use for diabetes, history of medication use for dyslipidemia, TC, HDL-C, LDL-C, HbA1c, hsCRP, and eGFR.

Table S5: Interaction and joint effects for exposures to TyG index and hypertension on stroke stratified by age

| Variable | Age <60 years | | |  | Age ≥60 years | | |
| --- | --- | --- | --- | --- | --- | --- | --- |
|  | No. of event | HR (95% CI) | P value |  | No. of event | HR (95% CI) | P value |
| Hypertension and TyG index |  |  |  |  |  |  |  |
| Without hypertension – Q1 of TyG index | 26 | Reference |  |  | 19 | Reference |  |
| Without hypertension – Q2 of TyG index | 30 | 1.13 (0.66–1.92) | 0.658 |  | 33 | 1.79 (1.01–3.16) | 0.045 |
| Without hypertension – Q3 of TyG index | 38 | 1.48 (0.88–2.49) | 0.136 |  | 34 | 2.11 (1.19–3.75) | 0.011 |
| Without hypertension – Q4 of TyG index | 24 | 0.81 (0.44–1.51) | 0.512 |  | 24 | 1.72 (0.89–3.33) | 0.107 |
| Hypertension – Q1 of TyG index | 21 | 2.38 (1.33–4.25) | 0.003 |  | 35 | 2.44 (1.39–4.28) | 0.002 |
| Hypertension – Q2 of TyG index | 26 | 2.36 (1.36–4.12) | 0.002 |  | 55 | 3.33 (1.96–5.66) | <0.001 |
| Hypertension – Q3 of TyG index | 56 | 3.54 (2.14–5.85) | <0.001 |  | 70 | 3.17 (1.86–5.40) | <0.001 |
| Hypertension – Q4 of TyG index | 64 | 2.14 (1.21–3.80) | 0.009 |  | 82 | 3.30 (1.85–5.87) | <0.001 |
| Synergy index | NA | 6.32 (0.85–46.89) | NA |  |  | 4.30 (2.13–8.65) | NA |
| HR for multiplicative | NA | 2.38 (1.33–4.25) | NA |  |  | 2.44 (1.39–4.28) | NA |
| SBP and TyG index |  |  |  |  |  |  |  |
| SBP <140 mmHg – Q1 of TyG index | 32 | Reference |  |  | 27 | Reference |  |
| SBP <140 mmHg – Q2 of TyG index | 37 | 1.07 (0.66–1.73) | 0.786 |  | 53 | 1.91 (1.20–3.06) | 0.007 |
| SBP <140 mmHg – Q3 of TyG index | 57 | 1.54 (0.98–2.43) | 0.063 |  | 56 | 1.93 (1.20–3.12) | 0.007 |
| SBP <140 mmHg – Q4 of TyG index | 54 | 1.03 (0.61–1.76) | 0.908 |  | 52 | 1.95 (1.13–3.36) | 0.016 |
| SBP ≥140 mmHg – Q1 of TyG index | 15 | 2.31 (1.24–4.28) | 0.008 |  | 27 | 2.15 (1.26–3.68) | 0.005 |
| SBP ≥140 mmHg – Q2 of TyG index | 19 | 2.47 (1.39–4.41) | 0.002 |  | 35 | 2.47 (1.48–4.12) | 0.001 |
| SBP ≥140 mmHg – Q3 of TyG index | 37 | 3.42 (2.05–5.70) | <0.001 |  | 48 | 2.76 (1.68–4.54) | <0.001 |
| SBP ≥140 mmHg – Q4 of TyG index | 34 | 1.68 (0.93–3.03) | 0.087 |  | 54 | 2.58 (1.50–4.43) | 0.001 |
| Synergy index | NA | 4.65 (1.99–10.89) | NA |  | NA | 3.77 (1.92–7.42) | NA |
| HR for multiplicative | NA | 2.31 (1.24–4.28) | NA |  | NA | 2.15 (1.26–3.68) | NA |
| DBP and TyG index |  |  |  |  |  |  |  |
| DBP <90 mmHg – Q1 of TyG index | 36 | Reference |  |  | 49 | Reference |  |
| DBP <90 mmHg – Q2 of TyG index | 43 | 1.09 (0.70–1.71) | 0.698 |  | 79 | 1.54 (1.07–2.22) | 0.020 |
| DBP <90 mmHg – Q3 of TyG index | 68 | 1.55 (1.01–2.38) | 0.046 |  | 85 | 1.55 (1.06–2.26) | 0.023 |
| DBP <90 mmHg – Q4 of TyG index | 62 | 1.01 (0.61–1.69) | 0.967 |  | 87 | 1.55 (0.99–2.41) | 0.055 |
| DBP ≥90 mmHg – Q1 of TyG index | 11 | 2.42 (1.22–4.78) | 0.011 |  | 5 | 1.31 (0.52–3.30) | 0.565 |
| DBP ≥90 mmHg – Q2 of TyG index | 13 | 2.36 (1.24–4.50) | 0.009 |  | 9 | 1.87 (0.91–3.83) | 0.089 |
| DBP ≥90 mmHg – Q3 of TyG index | 26 | 3.68 (2.15–6.31) | <0.001 |  | 19 | 2.46 (1.41–4.27) | 0.001 |
| DBP ≥90 mmHg – Q4 of TyG index | 26 | 1.60 (0.86–2.97) | 0.134 |  | 19 | 2.40 (1.31–4.38) | 0.005 |
| Synergy index | NA | 4.98 (1.92–12.89) | NA |  | NA | 1.96 (0.50–7.64) | NA |
| HR for multiplicative | NA | 2.42 (1.22–4.78) | NA |  | NA | 1.31 (0.52–3.30) | NA |

Abbreviations: CI, confidence interval; DBP, diastolic blood pressure; HR, hazard ratio; SBP, systole blood pressure; TyG, triglyceride-glucose.

^a^ All models were adjusted for gender, marital status, residence, education level, body mass index, smoking status, drinking status, diabetes, heart disease, dyslipidemia, kidney disease, history of medication use for diabetes, history of medication use for dyslipidemia, TC, HDL-C, LDL-C, HbA1c, hsCRP, and eGFR.

Table S6: Interaction and joint effects for exposures to TyG index and hypertension on stroke stratified by gender

| Variable | Male | | |  | Female | | |
| --- | --- | --- | --- | --- | --- | --- | --- |
|  | No. of event | HR (95% CI) | P value |  | No. of event | HR (95% CI) | P value |
| Hypertension and TyG index |  |  |  |  |  |  |  |
| Without hypertension – Q1 of TyG index | 23 | Reference |  |  | 22 | Reference |  |
| Without hypertension – Q2 of TyG index | 31 | 1.53 (0.89–2.63) | 0.128 |  | 32 | 1.20 (0.70–2.08) | 0.509 |
| Without hypertension – Q3 of TyG index | 33 | 1.92 (1.11–3.32) | 0.020 |  | 39 | 1.51 (0.88–2.59) | 0.133 |
| Without hypertension – Q4 of TyG index | 26 | 1.60 (0.86–2.96) | 0.138 |  | 22 | 0.81 (0.42–1.56) | 0.525 |
| Hypertension – Q1 of TyG index | 36 | 2.84 (1.67–4.82) | <0.001 |  | 20 | 1.75 (0.95–3.23) | 0.075 |
| Hypertension – Q2 of TyG index | 39 | 3.05 (1.80–5.17) | <0.001 |  | 42 | 2.48 (1.46–4.21) | 0.001 |
| Hypertension – Q3 of TyG index | 60 | 4.04 (2.42–6.73) | <0.001 |  | 66 | 2.53 (1.51–4.25) | <0.001 |
| Hypertension – Q4 of TyG index | 63 | 3.11 (1.76–5.49) | <0.001 |  | 83 | 2.21 (1.25–3.89) | 0.006 |
| Synergy index | NA | 5.08 (2.84–9.10) | NA |  | NA | 3.55 (0.78–16.24) | NA |
| HR for multiplicative | NA | 2.84 (1.67–4.82) | NA |  | NA | 1.75 (0.95–3.23) | NA |
| SBP and TyG index |  |  |  |  |  |  |  |
| SBP <140 mmHg – Q1 of TyG index | 31 | Reference |  |  | 28 | Reference |  |
| SBP <140 mmHg – Q2 of TyG index | 43 | 1.49 (0.93–2.38) | 0.094 |  | 47 | 1.31 (0.82–2.11) | 0.259 |
| SBP <140 mmHg – Q3 of TyG index | 53 | 1.98 (1.24–3.15) | 0.004 |  | 60 | 1.46 (0.91–2.34) | 0.112 |
| SBP <140 mmHg – Q4 of TyG index | 49 | 1.58 (0.92–2.69) | 0.095 |  | 57 | 1.21 (0.71–2.07) | 0.485 |
| SBP ≥140 mmHg – Q1 of TyG index | 28 | 2.73 (1.63–4.56) | <0.001 |  | 14 | 1.47 (0.76–2.81) | 0.250 |
| SBP ≥140 mmHg – Q2 of TyG index | 27 | 2.67 (1.57–4.52) | <0.001 |  | 27 | 1.95 (1.14–3.36) | 0.015 |
| SBP ≥140 mmHg – Q3 of TyG index | 40 | 3.48 (2.12–5.73) | <0.001 |  | 45 | 2.33 (1.40–3.86) | 0.001 |
| SBP ≥140 mmHg – Q4 of TyG index | 40 | 2.76 (1.59–4.79) | <0.001 |  | 48 | 1.61 (0.92–2.81) | 0.097 |
| Synergy index | NA | 4.83 (2.70–8.63) |  |  | NA | 2.37 (0.92–6.12) | NA |
| HR for multiplicative | NA | 2.73 (1.63–4.56) | NA |  | NA | 1.47 (0.76–2.81) | NA |
| DBP and TyG index |  |  |  |  |  |  |  |
| DBP <90 mmHg – Q1 of TyG index | 49 | Reference |  |  | 36 | Reference |  |
| DBP <90 mmHg – Q2 of TyG index | 54 | 1.21 (0.81–1.79) | 0.350 |  | 68 | 1.42 (0.94–2.14) | 0.095 |
| DBP <90 mmHg – Q3 of TyG index | 70 | 1.63 (1.10–2.40) | 0.015 |  | 83 | 1.47 (0.97–2.22) | 0.071 |
| DBP <90 mmHg – Q4 of TyG index | 65 | 1.32 (0.83–2.10) | 0.239 |  | 84 | 1.22 (0.75–1.98) | 0.426 |
| DBP ≥90 mmHg – Q1 of TyG index | 10 | 2.05 (1.03–4.06) | 0.041 |  | 6 | 1.86 (0.78–4.42) | 0.162 |
| DBP ≥90 mmHg – Q2 of TyG index | 16 | 2.63 (1.48–4.67) | 0.001 |  | 6 | 1.43 (0.60–3.42) | 0.416 |
| DBP ≥90 mmHg – Q3 of TyG index | 23 | 3.18 (1.88–5.36) | <0.001 |  | 22 | 3.03 (1.73–5.29) | <0.001 |
| DBP ≥90 mmHg – Q4 of TyG index | 24 | 2.54 (1.41–4.57) | 0.002 |  | 21 | 1.66 (0.89–3.08) | 0.110 |
| Synergy index | NA | 3.59 (1.53–8.39) |  |  | NA | 3.38 (1.04–11.02) | NA |
| HR for multiplicative | NA | 2.05 (1.03–4.06) |  |  | NA | 1.86 (0.78–4.42) | NA |

Abbreviations: CI, confidence interval; DBP, diastolic blood pressure; HR, hazard ratio; SBP, systole blood pressure; TyG, triglyceride-glucose.

^a^ All models were adjusted for age, marital status, residence, education level, body mass index, smoking status, drinking status, diabetes, heart disease, dyslipidemia, kidney disease, history of medication use for diabetes, history of medication use for dyslipidemia, TC, HDL-C, LDL-C, HbA1c, hsCRP, and eGFR.

**Table S7: Mediated effects by hypertension on the associations of TyG index with stroke**  **in subpopulations of 8 693 participants with complete data ^a^**

|  | Effects of TyG index with stroke (95% CI) | P value |
| --- | --- | --- |
| Mediation: Hypertension |  |  |
| Total effects | -14.15 (-34.53 to -1.91) | 0.006 |
| Direct effects | -11.60 (-30.44 to -0.25) | 0.044 |
| Mediated effects | -2.55 (-4.56 to -1.21) | <0.001 |
| Proportion mediated by hypertension | 18.0% |  |
| Mediation: SBP |  |  |
| Total effects | -15.13 (-34.05 to -2.21) | 0.012 |
| Direct effects | -13.10 (-31.17 to -0.74) | 0.026 |
| Mediated effects | -2.04 (-3.50 to -0.91) | <0.001 |
| Proportion mediated by SBP | 13.5% |  |
| Mediation: DBP |  |  |
| Total effects | -14.34 (-32.76 to -1.74) | 0.012 |
| Direct effects | -12.85 (-30.34 to -0.75) | 0.030 |
| Mediated effects | -1.49 (-2.73 to -0.57) | <0.001 |
| Proportion mediated by DBP | 10.4% |  |

Abbreviations: CI, confidence interval; DBP, diastolic blood pressure; SBP, systole blood pressure; TyG, triglyceride-glucose.

^a^ All models were adjusted for age, gender, marital status, residence, education level, body mass index, smoking status, drinking status, diabetes, heart disease, dyslipidemia, kidney disease, history of medication use for diabetes, history of medication use for dyslipidemia, TC, HDL-C, LDL-C, HbA1c, hsCRP, and eGFR.

**Table S8: Mediated effects by hypertension at Wave 2 on the associations of TyG index with stroke ^a^**

|  | Effects of TyG index with stroke (95% CI) | P value |
| --- | --- | --- |
| Mediation: Hypertension at Wave 2 |  |  |
| Total effects | -19.51 (-47.23 to -2.59) | 0.018 |
| Direct effects | -15.60 (-41.67 to -0.19) | 0.050 |
| Mediated effects | -3.91 (-7.36 to -1.85) | <0.001 |
| Proportion mediated by hypertension | 20.0% |  |
| Mediation: SBP at Wave 2 |  |  |
| Total effects | -19.08 (-47.47 to -2.83) | 0.006 |
| Direct effects | -16.73 (-42.94 to -1.46) | 0.020 |
| Mediated effects | -2.35 (-4.39 to -1.05) | <0.001 |
| Proportion mediated by SBP | 12.3% |  |
| Mediation: DBP at Wave 2 |  |  |
| Total effects | -18.69 (-46.29 to -2.67) | 0.006 |
| Direct effects | -16.44 (-41.93 to -1.35) | 0.018 |
| Mediated effects | -2.25 (-4.13 to -1.04) | <0.001 |
| Proportion mediated by DBP | 12.0% |  |

Abbreviations: CI, confidence interval; DBP, diastolic blood pressure; SBP, systole blood pressure; TyG, triglyceride-glucose.

^a^ All models were adjusted for age, gender, marital status, residence, education level, body mass index, smoking status, drinking status, diabetes, heart disease, dyslipidemia, kidney disease, history of medication use for diabetes, history of medication use for dyslipidemia, TC, HDL-C, LDL-C, HbA1c, hsCRP, and eGFR.

**Table S9: Mediated effects by hypertension on the associations of TyG index with stroke stratified by gender ^a^**

|  | Male | |  | Female |  |
| --- | --- | --- | --- | --- | --- |
|  | Effects of TyG index with stroke (95% CI) | P value |  | Effects of TyG index with stroke (95% CI) | P value |
| Mediation: Hypertension |  |  |  |  |  |
| Total effects | -20.39 (-71.72 to 3.26) | 0.144 |  | -10.19 (-27.70 to 0.36) | 0.062 |
| Direct effects | -16.83 (-64.53 to 4.90) | 0.258 |  | -8.23 (-25.26 to 1.51) | 0.132 |
| Mediated effects | -3.56 (-8.89 to -0.98) | 0.002 |  | -1.96 (-3.88 to -0.82) | <0.001 |
| Proportion mediated by hypertension | 17.5% |  |  | 19.2% |  |
| Mediation: SBP |  |  |  |  |  |
| Total effects | -21.99 (-75.03 to 2.50) | 0.114 |  | -10.96 (-30.04 to -0.10) | 0.044 |
| Direct effects | -18.47 (-68.32 to 3.84) | 0.198 |  | -10.03 (28.09 to 0.43) | 0.066 |
| Mediated effects | -3.52 (-8.15 to -0.94) | 0.002 |  | -0.93 (-1.95 to -0.29) | 0.044 |
| Proportion mediated by SBP | 16.0% |  |  | 8.5% |  |
| Mediation: DBP |  |  |  |  |  |
| Total effects | -20.99 (-72.45 to 2.81) | 0.130 |  | -10.56 (-28.31 to 0.14) | 0.052 |
| Direct effects | -18.47 (-67.05 to 3.60) | 0.190 |  | -9.92 (-27.00 to 0.39) | 0.058 |
| Mediated effects | -2.52 (-6.24 to -0.39) | 0.010 |  | -0.64 (-1.49 to -0.09) | 0.012 |
| Proportion mediated by DBP | 12.0% |  |  | 6.1% |  |

Abbreviations: CI, confidence interval; DBP, diastolic blood pressure; SBP, systole blood pressure; TyG, triglyceride-glucose.

^a^ All models were adjusted for age, marital status, residence, education level, body mass index, smoking status, drinking status, diabetes, heart disease, dyslipidemia, kidney disease, history of medication use for diabetes, history of medication use for dyslipidemia, TC, HDL-C, LDL-C, HbA1c, hsCRP, and eGFR.

**Table S10: Mediated effects by hypertension on the associations of TyG index with stroke stratified by age ^a^**

|  | Age <60 years | |  | Age ≥60 years |  |
| --- | --- | --- | --- | --- | --- |
|  | Effects of TyG index with stroke (95% CI) | P value |  | Effects of TyG index with stroke (95% CI) | P value |
| Mediation: Hypertension |  |  |  |  |  |
| Total effects | -7.10 (-29.45 to 4.10) | 0.380 |  | -26.03 (-67.69 to -3.20) | 0.006 |
| Direct effects | -5.14 (-26.07 to 5.30) | 0.570 |  | -22.31 (-60.52 to -1.21) | 0.022 |
| Mediated effects | -1.96 (-4.20 to -0.58) | <0.001 |  | -3.71 (-7.79 to -1.37) | <0.001 |
| Proportion mediated by hypertension | 27.6% |  |  | 14.3% |  |
| Mediation: SBP |  |  |  |  |  |
| Total effects | -7.59 (-30.76 to 3.86) | 0.350 |  | -26.64 (-69.35 to -3.37) | 0.006 |
| Direct effects | -5.72 (-26.84 to 4.77) | 0.510 |  | -24.81 (-64.32 to -2.52) | 0.012 |
| Mediated effects | -1.87 (-3.93 to -0.64) | <0.001 |  | -1.83 (-4.20 to -0.44) | 0.004 |
| Proportion mediated by SBP | 24.6% |  |  | 6.9% |  |
| Mediation: DBP |  |  |  |  |  |
| Total effects | -7.50 (-30.73 to 3.91) | 0.374 |  | -25.08 (-63.28 to -2.98) | 0.008 |
| Direct effects | -5.96 (-27.15 to 4.73) | 0.496 |  | -24.04 (-61.90 to -2.61) | 0.010 |
| Mediated effects | -1.53 (-3.46 to -0.34) | <0.001 |  | -1.04 (-2.63 to -0.10) | 0.016 |
| Proportion mediated by DBP | 20.4% |  |  | 4.1% |  |

Abbreviations: CI, confidence interval; DBP, diastolic blood pressure; SBP, systole blood pressure; TyG, triglyceride-glucose.

^a^ All models were adjusted for gender, marital status, residence, education level, body mass index, smoking status, drinking status, diabetes, heart disease, dyslipidemia, kidney disease, history of medication use for diabetes, history of medication use for dyslipidemia, TC, HDL-C, LDL-C, HbA1c, hsCRP, and eGFR.
